# Still searching for the female norm: a GLUT of knowledge remains to be revealed

**DOI:** 10.1113/EP091204

**Published:** 2023-05-02

**Authors:** Vicky Taylor

**Affiliations:** ^1^ School of Life, Health & Chemical Sciences (LHCS) The Open University, Walton Hall Milton Keynes Buckinghamshire UK

**Keywords:** enteroplasticity, female, gastrointestinal tract, lactation, ovarian cycle, pregnancy, reproduction

1

For something that takes place so regularly – between every 21 and 35 days – and affects up to one half of the human population, why is it that we still have only limited knowledge about other associated internal metabolic fluctuations that are taking place throughout this dynamic time known as the ovarian cycle? Is it simply down to the historical avoidance of undertaking research in female subjects? Or is it due to less appreciation of the inter‐relationships between seemingly more distant physiological structures and systems that could also be involved? In this issue of *Experimental Physiology*, by investigating female mouse small intestine gut tissues *ex vivo* that were taken during clearly identified stages of the murine ovarian cycle, Overduin et al. (2023) have tackled this lack of information head on. Their efforts were rewarded in isolating subtle changes that potentially will be even more revealing when teamed with other research findings. The authors applied a refined Ussing chamber methodology to address their research questions directly, and in doing so found fluctuations in active sodium glucose‐cotransporter 1 (SGLT1)‐mediated glucose transport in the gut, with reductions in glucose uptake during the transition towards ovulatory status, in ad libitum‐fed mice. At first glance, this may not seem particularly startling, but it allows more appropriate interpretation of existing research findings about glucose control to be considered in females. These alterations in measured induced d‐glucose uptake contribute some mechanistic support towards developing biological explanations for the long‐held observations in some female mammals of lower food intake (including humans: Virgil et al., 2022) and increased energy expenditure just prior to the behavioural expression of oestrus and the physical event of ovulation. Moreover, this adds more impetus to the new drive to fund, perform and publish research with equal representation of male and female subjects and/or physiology (e.g., US National Institute of Health, NIH; UK Medical Research Council, MRC) to address the imbalance in the underlying knowledge base. Improved understanding will aid the design of future studies to answer other long‐standing questions about female‐specific physiology and allow the translation of new findings into precision medicine to benefit all (Karp & Reavey, 2019).

A regular supply of glucose is critical for survival but its continuous circulation within the body in excess can be toxic. Tightly controlled whole body glucose regulation allows normal functioning to take place efficiently and its effective management is protective against the many negative effects, including damage to multiple tissues that can occur within a dysregulated system. The major interacting promoters of postprandial insulin secretion work in different ways to regulate blood glucose concentrations after a meal. Glucose‐dependent insulinotropic hormone (GIP) is secreted from K‐cells in the duodenum and proximal jejunum of the small intestine, and glucagon‐like peptide 1 (GLP‐1) from L‐cells in the distal ileum and large intestine/colon. The integration of the effects of these gut hormones governs the absorption of glucose into intestinal cells (mainly via SGLT1) and its subsequent entry into the circulation (via GLUT2; Koepsell, 2020) which elevates the pre‐existing blood glucose concentration, which then feeds back. SGLT1 is diurnally expressed in L and K cells and involved in d‐glucose‐dependent stimulation of enterohormone secretion. These various steps and changes may be differentially acting on a number of different regulatory control axes (Zhu et al., 2022) and appropriate coordination of the sequencing of each event tightly adjusts the fate of ingested glucose to immediate metabolic requirements: use or storage. Fine balancing of the entire system controls any excessive hyperglycaemia/insulinaemia and similarly avoids extreme hypoglycaemia, without which there would eventually be toxic effects on multiple tissues, or coma then death. But beyond these extremes and within normal ranges, there are temporary alterations and/or homeorhetic adjustments that take place to serve shorter term overriding functions, often related to reproductive events. Overduin et al. may have uncovered some evidence for one such ‘normal’ change occurring just prior to oestrus. This naturally raises further questions about what other related factors might also be altered at this critical time, and furthermore poses the more obvious question, why? Or alternatively and maybe more importantly, what happens if this ‘normal’ situation does *not* occur prior to ovulation?

These observations of attenuated d‐glucose uptake at a definitive time in the mouse ovarian cycle tie in with some earlier unexpected findings associated with these integrated regulatory control mechanisms. GLP‐1 in fed plasma and descending colon tissue was observed to increase prior to oestrus in rats (Johnson et al., 2017). Combined with an associated observation of lowest food intake at oestrus, these increases in GLP‐1 were interpreted to contribute to the temporary anorexigenic tone at this time, as it normally acts as an incretin. Overduin et al.’s findings were obtained using tissues from ad libitum‐fed mice and due to their being group‐housed, they were not able to relate them to any food intake changes. Additional, more intensive studies are therefore needed to unravel any other potentially different influences on uptake of mixed‐meal content, as opposed to d‐glucose on its own (with a range of concentrations). However it is timely to consider the implications of these combined preliminary findings and important to clearly establish the norms of equivalent control mechanisms operating within the human ovarian cycle. This is because the incretin properties of analogues of GLP‐1, one signal within this complex regulatory system, are already being used clinically to treat type II diabetes, and similar pharmaceutical products are now being approved more widely for use to achieve body mass reductions (Vigil et al., 2022), increasingly in females of reproductive age. What cannot be gained from Overduin et al.’s small study is *why* a reduction in glucose uptake from the small intestine might be characteristic of or biologically important in the short pre‐ovulatory period, or even whether the same occurs in humans. However it is necessary to continue to improve our understanding of what the optimal nutritional and metabolic environments are to ensure that healthy gametes are produced, in advance of potential conception.

Turning to the second main finding in the current study, Overduin et al. pinpointed that the active d‐glucose uptake peak values measured occurred in a specific gut region: within the distal jejunum. The authors acknowledge that more work is needed to establish the extent to which food intake and diurnal SGLT1 changes, gastric emptying, and/or multiple hormone effects are involved in influencing the changes observed. However, the location itself is notable as it is already well established that physical adaptations can occur in response to the high energetic demands of pregnancy and lactation in rodents (Naya et al., 2007). Increases take place in the absorptive capacity of the maternal small intestine and the most dramatic mass gains are found in duodenum and jejunum, where the majority of breakdown and absorption occurs. Future studies employing the same Ussing chamber methodology as the current ovarian study but using pregnant and maternal gut sections are proposed. However as these are not short transient ovarian stages but more prolonged reproductive states, with greater energetic demands involving up to 400% food intake increases in rodents, there can be concomitant extensive gastrointestinal structural modifications. These involve increases in tissue masses and overall gut length – so to be informative, any measured uptake rates will need to be calculated and reported as mucosal and/or intestinal mass and length specific. These adaptive changes involve a complicated array of epithelial cell adjustments – number, size, type/differentiation status, turnover, villus height, microvillus length, variable/different nutrient transporters (Koepsell, 2020), any or all of which can affect calculations or comparisons of glucose uptake rate. This means that average value changes, without accounting for other alterations, will not accurately reflect the total summed glucose uptake that occurs or is possible within the entire gastrointestinal area of interest. Hammond (1997) has developed a useful framework that could be used as a starting point to aid the design of future studies in this area. She calculated and plotted the summed glucose uptake, representing systemic capacity of the blood circulation, for virgin mice, pregnant mice, and lactating mice supporting a large litter of 14 pups. Below this, another plot represents their expected food intake requirements (equivalent to intestinal glucose load) thereby providing an indication, via the differences revealed, of the available ‘excess’ capacity that could be employed during these three reproductive conditions, each with varying energetic requirements. The resulting differences illustrate how quickly any existing excess capacity could be used up, particularly in reaching peak lactation, and clearly shows why intestinal mass/length increases are essential to maintain sufficient maternal nutrient uptake rapidly enough to produce the required milk to fulfil lactation demands.

Whilst much research attention is generally focused on investigating metabolic dysfunction, more straightforward physiological studies such as this one should be encouraged. This will establish the normal adaptive adjustments to temporary female reproductive conditions, and carefully distinguish them from aberrant conditions. The outcomes of such future studies will establish to what extent glucoregulatory control is temporarily altered in normal uncomplicated ovarian cycles, pregnancy and lactation, to differentiate them from conditions that may need intervention or management. Similarly, if there are any equivalent enteroplastic structural changes during human pregnancy and lactation to those documented in other mammalian species, are they only temporary and revert back after these events have taken place, or do they remain and contribute to enhanced nutrient harvesting during future reproductive events and/or in obesogenic environments? Either way, understanding more about the mechanisms governing specific, temporary, physiological events could provide additional avenues to explore how to reverse or resolve other more prolonged or intractable metabolic dysfunctions to minimise their associated health risks for all.

## AUTHOR CONTRIBUTIONS

Sole author.

## CONFLICT OF INTEREST

The author declares no conflicts of interest.

## FUNDING INFORMATION

No funding was received for this work.

## References

[eph13370-bib-0001] Hammond, K. (1997). Adaptation of the maternal intestine during lactation. Journal of Mammary Gland Biology and Neoplasia, 2(3), 243–252. 10882308 10.1023/a:1026332304435

[eph13370-bib-0002] Johnson, M. L. , Saffrey, M. J. , & Taylor, V. J. (2017). Glucagon‐like peptide‐1 (GLP‐1) increases in plasma and colon tissue prior to estrus and circulating levels change with increasing age in reproductively competent Wistar rats. Peptides, 90, 55–62. 28237410 10.1016/j.peptides.2017.02.010

[eph13370-bib-0003] Karp , & Reavey (2019). Sex bias in preclinical research and an exploration of how to change the status quo. British Journal of Pharmacology, 176(21), 4107–4118. 30418665 10.1111/bph.14539 PMC6877896

[eph13370-bib-0004] Koepsell, H. (2020). Glucose transporters in the small intestine in health and disease. Pflügers Archiv – European Journal of Physiology, 472(9), 1207–1248. 32829466 10.1007/s00424-020-02439-5 PMC7462918

[eph13370-bib-0005] Naya, D. E. , Karasov, W. H. , & Bozinovic, F. (2007). Phenotypic plasticity in laboratory mice and rats: A meta‐analysis of current ideas on gut size flexibility. Evolutionary Ecology Research, 9, 1363–1374.

[eph13370-bib-0006] Overduin, T. S. , Wardill, H. R. , Young, R. L. , Page, A. , & Gatford, K. L. (2023). Active glucose transport varies by small intestinal region and estrous cycle stage in mice. Experimental Physiology, 108(6), 865–873. 37022128 10.1113/EP091040 PMC10988461

[eph13370-bib-0007] Vigil, P. , Meléndez, J. , Petkovic, G. , & Pablo Del Río, J. (2022). The importance of estradiol for body weight regulation in women. Frontiers in Endocrinology, 13, 951186. 36419765 10.3389/fendo.2022.951186 PMC9677105

[eph13370-bib-0008] Zhu, H. , Cai, H. , Wang, X. , Chen, T. , Zhen, C. , Zhang, Z. , Ruan, X. , & Li, G. (2022). Sodium‐glucose co‐transporter 1 (SGLT1) differentially regulates gluconeogenesis and GLP‐1 receptor (GLP‐1R) expression in different diabetic rats: A preliminary validation of the hypothesis of “SGLT1 bridge” as an indication for “surgical diabetes”. Annals of Translational Medicine, 10(8), 481. 35571394 10.21037/atm-22-1769 PMC9096370

